# Current Advances in Immunotherapy for Acute Leukemia: An Overview of Antibody, Chimeric Antigen Receptor, Immune Checkpoint, and Natural Killer

**DOI:** 10.3389/fonc.2019.00917

**Published:** 2019-09-19

**Authors:** Yufeng Shang, Fuling Zhou

**Affiliations:** Department of Hematology, Zhongnan Hospital of Wuhan University, Wuhan, China

**Keywords:** immunotherapy, acute myeloid leukemia, acute lymphoblastic leukemia, antibody-drug conjugate, bispecific antibody, chimeric antigen receptor, immune checkpoint

## Abstract

Recently, due to the application of hematopoietic stem cell transplantation and small molecule inhibitor, the survival of acute leukemia is prolonged. However, the 5 year survival rate remains low due to a high incidence of relapse. Immunotherapy is expected to improve the prognosis of patients with relapsed or refractory hematological malignancies because it does not rely on the cytotoxic mechanisms of conventional therapy. In this paper, the advances of immunotherapy in acute leukemia are reviewed from the aspects of Antibody including Unconjugated antibodies, Antibody-drug conjugate and Bispecific antibody, Chimeric Antigen Receptor (CARs), Immune checkpoint, Natural killer cells. The immunological features, mechanisms and limitation in clinic will be described.

## Introduction

Acute leukemia is a kind of hematological malignancy with high mortality and poor prognosis and it requires a complex and highly diversified treatment because of its wide prognostic heterogeneity. Although traditional treatments such as chemotherapy and hematopoietic stem cell transplantation make patients obtain complete remission, patients will eventually develop relapse and resistance, leading to disease progression. The average cure rate was only 35% in adult acute lymphoblastic leukemia (ALL) patients and 40% in aged ≤65 years acute myeloid leukemia (AML) patients relying on traditional treatment (1, 2). Allogenic hematopoietic stem cell transplantation (allo-HSCT) increases the cure rate of ALL to about 50%, and offers significant relapse-free survival and overall survival benefits for intermediate- and poor-risk AML, and also cures a proportion of patients with refractory/relapsed AML, but transplantation related mortality (TRM) has reached 20–30% (1, 3–6). In fact, allo-HSCT is also a form of immunotherapy through graft-vs.-leukemia (GvL) effects by the adoptively transferred donor T-cells, which is seen as the first successful clinical application of immunotherapy. Based on GvL effects, the non-myeloablative allogeneic stem cell transplantation has been developed, thus reducing TRM (7). Some anti-AML drugs such azacytidine and sorafenib have shown to promote GvL effects without increasing the risk of graft-vs.-host disease (GVHD) by regulating immune cells (8–10). Besides, donor lymphocyte infusions (DLI) as a form of pure immunotherapy not only could prevent recurrence after transplantation but also have cured a number of acute leukemia patients (11). Therefore, immune related intervention plays a key role in the treatment of acute leukemia.

**Figure 1:**
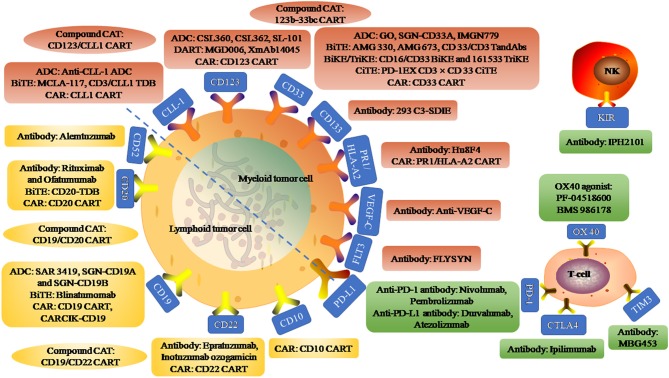
An overview of immunotherapy targets for acute myeloid leukemia and acute lymphoblastic leukemia. Myeloid tumor cell surface antigen targets include CLL-1, CD123, CD33, CD133, PR1/HLA-A2, VEGF-C, and FLT3. Anti-CLL-1 ADC, an antibody-drug conjugate (ADC) targeting CLL-1 that binds to pyrrole diazepine dimer. MCLA-117 is a full-length human IgG1 Bi-Specific T-cell Engagers (BiTE) targeting CLL-1 and CD3. CD3/CLL1 TDB, a CD3 T-cell-dependent bispecific (TDB) full-length humanized IgG1 antibody targeting CLL1. CSL360 and CSL362 are recombinant chimeric antibody targeting CD123 and fully humanized anti-CD123 antibody, respectively. SL-101, an CD123 ADC fused to Pseudomonas exotoxin A. MGD006 is a bispecific CD3xCD123 dual-affinity re-targeting (DART) molecule. XmAb14045, a structured anti-CD123 T cell recruitment antibody. GO, SGN-CD33A, and IMGN779 are CD33 ADC binding to N-acetylgamma-erythromycin, pyrrolidone dimer and DNA alkylation activity, respectively. AMG 330 is a CD33/CD3 BiTE and AMG 673 is the second CD33/CD3 BiTE. CD33/CD3 TandAbs is a directional tandem tetravalent bispecific antibody. CD16/CD33 BiKE is a Bi-Specific Killer Engagers (BiKE) targeting CD16 and CD33, and 161533 TriKE is a Tri-Specific Killer Engagers (TiKE) including a modified IL-15 crosslinking agent between CD 16 and CD 33 single-chain Fv fragments. PD-1EX with CD3xCD33 BiTE, fused the PD-1 extracellular domain (PD-1EX) with CD3xCD 33 BiTE. Hu8F4, a T-cell receptor-like monoclonal antibody targeting PR1/HLA-A2. PR1/HLA-A2 CART, PR1/HLA-A2-specific CART (h8F4-CAR-T cells), containing the scFv of h8F4 fused to CD3 zeta chain through the co-stimulatory domain of CD28. Anti-VEGF-C, an antibody targeting vascular endothelial growth factor C to reduce expansion and enhance differentiation. FLYSYN and 293 C3-SDIE are antibodies targeting FLT3 and CD133. Compound CART include 123b-33bc CART simultaneously targeting CD123 and CD33, and CD123/CLL1 CART simultaneously targeting CD123 and CLL1. Lymphoid tumor cell surface antigen targets include CD52, CD20, CD19, CD22, CD10. Rituximab and Ofatumumab are antibodies targeting CD20. CD20-TDB, is a CD3 TDB full-length humanized immunoglobulin G1 molecule targeting CD20. SAR 3419, SGN-CD19A, and SGN-CD19B are CD 19 ADC that binds to Maytansin, Monoinositol F and Pyrrolidine diazepine, respectively. Blinatumomab, a BiTE with dual specificity for CD19 and CD3. CARCIK-CD19, a cytokine-induced killer (CIK) cells transfected with the transposon CD19CAR. Epratuzumab and Inotuzumab ozogamicin are unconjugated human antibody targeting CD22 and humanized anti-CD 22 ADC, respectively. Alemtuzumab, an anti-CD52 antibody. Compound CART include CD19/CD20 CART simultaneously targeting CD19 and CD20, and CD19/CD22 CART simultaneously targeting CD19 and CD22. Immune targets include inhibitory receptors programmed cell death protein 1 (PD1), cytotoxic T-lymphocyte antigen 4 (CTLA4), T-cell immunoglobulin and mucin-domain containing-3 (TIM3), and stimulatory receptors OX40 on T-cell subsets and their ligands (PD-L1, PD-L2, B7, and OX40L) on AML blasts. IPH2101, anti antibody targeting KIR on NK cell surface.

In recent years, the emergence of immunotherapy has brought hope to hematological malignancies. Here, we reviewed the research progress of immunotherapy in acute leukemia. Immunotherapy mainly includes targeting AML/ALL cell surface antigen (such as CD33, CD123, CLL-1 on AML and CD20, CD19, CD52 on ALL), relieving T/NK cell immunosuppression (such as PD1/PD-L1, CTLA4), using immunopotentiator (such as OX40 agonist) and adoptive cell therapy (such as CART), etc. This study focused on the naked monoclonal antibodies (mAbs), antibody-drug conjugate (ADC), bispecific T cell engager (BiTE), chimeric antigen receptor (CAR) T/NK cell, immune checkpoint inhibitor/immune agonist, etc. An overview of immunotherapy targets for AML and ALL is shown in Figure 1.

## Unconjugated Antibodies/Antibody-Drug Conjugate (ADC)

Monoclonal antibody binding to leukemia target antigen can lead to direct apoptosis, complement-dependent cytotoxicity (CDC) and antibody-dependent cell-mediated cytotoxicity (ADCC) (12). Potential targets for the treatment of AML include CD33, CD45, CD96, CD123, CD135, C-type lectin-like molecule-1 (CLL1), and FLT3 and so on, among of which CD33 and CD123 also expressed on hematopoietic stem cells (HSCs) while CLL1 absent on megakaryocytic progenitor cells and CD34+/CD38-HSCs (13). The potential targets for ALL include CD19, CD20, CD22, and CD52 *etc*. (12, 14–16). Antibody therapy include unconjugated antibodies and ADC. ADC can deliver a more potent cytotoxic payload specifically targeting leukemia cells accompanying the conjugated monoclonal antibody if a surface marker to internalize upon binding, and maintain lower systemic concentrations to enhance the effectiveness of induction chemotherapy.

### Anti-CD33 Antibody

CD33 is a transmembrane cell surface receptor and a member of the sialic acid-binding immunoglobulin (Ig)-like lectins, containing two conserved tyrosine-based inhibitory signaling motifs for recruitment and activation of the tyrosine phosphatases SHP-1 and SHP-2 or suppressor of cytokine signaling 3 (17). CD33 widely expressed on AML blasts about 90%, and on normal multi-potent myeloid precursor cells, unipotent myeloid colony-forming cells, and maturing myeloid cells, monocytes, peripheral granulocytes and so on (18).

#### Gemtuzumab Ozogamicin (GO)

GO is the first recombinant humanized ADC targeting CD33, which is linked to N-acetyl-γ-carramycin delivering a DNA-damaging calicheamicin derivative. In 2000, FDA approved GO for recurrent CD 33 + AML aged 60 years or older (19). But in the confirmed III phase clinical study of SWOG S 0106, the addition of GO to induction therapy or after consolidation did not improve the CR rate, DFS or OS, and the mortality rate of GO group increased, so GO automatically withdrew from the market (20). In subsequent studies, it was confirmed that adding GO benefits OS in patients with good cytogenetics and reduces the risk of recurrence (21, 22). Although GO has shown a narrow therapeutic window in early clinical studies, recent reports have proved that the improved dose regimen of GO combined with induced chemotherapy was safe and provided significant survival value for AML patients. Cathy C Zhang has shown that low dose GO combined with induced chemotherapy was able to remove minimal residual diseases more effectively, including LICs, thus achieving a more lasting remission effect and improving the survival of model mice (23). In the randomized phase III EORTC-GIMEMA AML-19 study, for the elderly patients who were not suitable for intensive chemotherapy in the initial treatment of AML, the use of low dose GO was better than the best support therapy in improving OS (4.9 months vs. 3.6 months; HR = 0.69; 95% CI, 0.53~0.90; *p* = 0.005). The side effects in the two groups were similar and the toxicity was controllable (24). The randomized phase III ALFA 0701 trial showed that adding reduced or segmented doses of GO to standard first-line chemotherapy significantly improved the event-free survival rate in primary AML patients (13.6 months vs. 8.8 months; HR = 0.68; 95% CI, 0.51~0.91) (25). Based on the above two studies, on September 1, 2017, FDA approved GO for the treatment of CD 33 positive adult AML (26). The expression of CD 33 is considered to be a predictor of the efficacy of GO in adult AML (27). The clinical study of stage III in Children's Oncology Group Trial AAML0531 showed that CD 33 splicing polymorphism determined the response of GO to primary AML, especially for CC genotype patients, to conclude that the recurrence rate of GO group was significantly lower than that of non-GO group (26% vs. 49%, *p* < 0.001) (28). In order to improve the therapeutic response, GO combined with epigenetics therapy is also being studied. For example, in clinical trials of GO combined with histone deacetylase inhibitor Vorinostat and DNA methyltransferase I inhibitor Azacitidine in elderly patients with relapsed or refractory (r/r)AML phase I/II, the ORR was as high as 41.9% in patients receiving maximum tolerated dose (29).

#### SGN-CD33A

An ADC targeting CD33 conjugated to a highly potent, synthetic DNA cross-linking pyrrolobenzodiazepine (PBD) dimer via a protease-cleavable linker leading to cell death. SGN-CD33A overcome some limitations of GO, such as the non-uniform conjugation of the toxin with the antibody, the drug's relatively slow internalization kinetics, and toxin extrusion via drug transporters (30). In combination with hypomethylating agents (HMAs) treating AML patients, the remission rate was significantly increased, but the hematological toxicity was increased at the same time (31). SGN-CD33A has been reported to cause liver damage, especially sinusoidal syndrome, which has been terminated by FDA. Dose-adjusted SGN-CD33A can reduce adverse reactions. Phase I clinical studies are under way to assess its toxicity and efficacy (32).

#### IMGN779

Another preclinical studies of anti-CD-33 antibodies, a conjugate with DNA alkylation activity, possessing good antitumor effects in mouse model and AML cell lines, whose cytotoxic activity involved DNA damage, cell-cycle arrest, and apoptosis (33). In order to improve the specificity of target cells for AML, alternative target antigens, such as CD25, FLT3 in the early clinical stage, are being studied (34, 35).

### Anti-CD123 Antibody

Interleukin-3 (IL-3) receptor α (CD123) is not only constitutively expressed on normal committed hematopoietic progenitor cells, but also highly expressed in AML blasts, including leukemic stem cells (LSCs) (36, 37). IL-3 is an activated T cell product that bridges innate and adaptive immunity and contributes to several immunopathologies.

#### CSL360

CSL 360 is a recombinant chimeric immunoglobulin G 1 anti CD123 monoclonal antibody, which can recognize the CD123(+)/CD131(–) phenotype expressed by LSCs exhibiting anti leukemia activity by neutralizing IL-3 to prevent IL-3 binding to its receptor (38, 39). *In vitro*, blockade of CD123 in AML cell lines abolishes proliferation induced by IL-3 (39, 40). Stage I clinical trials proved its safety, but it did not show definite anti-leukemia activity in most patients with AML (38). CSL360 modified with diethylenetriaminepentaacetic acid for complexing 111In and 13-mer nuclear translocation sequence peptides to enable nuclear importation in LSCs for Auger electron radioimmunotherapy of AML was studied by Leyton et al. (41).

#### CSL362

The second generation humanized, affinity-matured and Fc-engineered anti CD123 monoclonal antibody, induces stronger ADCC by high affinity to CD16 of NK cells (42, 43). Several studies have proved that it has good antitumor activity in mice and can prolong the survival time of mice (42, 44). Xie et al. reported a case of leukemia cell resistant to autologous ADCC. After allogeneic hematopoietic stem cell transplantation, CSL362 combined with donor-derived NKs effectively dissolved these cells, suggesting that CSL362 can be used as a promising therapy for chemotherapy and transplantation (45). Another antibody SL-101, which is a novel antibody-conjugate comprising an anti-CD123 single-chain Fv fused to *Pseudomonas exotoxin A*, is also in the preclinical study (46).

### Anti-CLL-1 Antibody

CLL-1 is a type II transmembrane glycoprotein of myeloid differentiation antigen, which is highly expressed in AML blasts and LSCs (13, 47). Unlike other targets such as CD33 and CD123, CLL-1 is not expressed on normal HSCs, which can ensure hematopoietic recovery, therefore provide a potential therapeutic target for AML treatment (48).

#### CLL-1 ADC

CLL-1 ADC is an anti CLL-1 antibody that contains a highly potent PBD dimer DNA alkylating and cross-linking agent, conjugated through a novel self-immolative disulfide linker, which is very effective in eliminating xenografts in mice or monkey leukemia cells and has little toxicity. It is expected to be an effective and safe treatment for AML (49).

Other antibodies against AML include Hu8F4 (T-cell receptor-like monoclonal antibody binding PR1/HLA-A2 complex on the surface of AML cells), VEGF-C (Vascular Endothelial Growth Factor-C) antibody (50–53).

Selected trials of antibody directed therapy for AML has been completed or is ongoing as shown in Table 1.

**Table 1:** Selected completed or ongoing trials of antibody directed therapy for AML.

**Target**	**Biological**	**Clinical trials**	**Purpose**	**Patients**	**Intervention/Treatment**	**Status**	***N***	**Phase**	**Results**
CD33	GO	NCT00085709	Efficacy	Newly diagnosed younger AML.	Induction 7+3 +/– GO	Completed	637	III	The CR rate was 69% for DA+GO group and 70% for DA group (*P* = 0.59). DFS was not improved with GO
		NCT00372593	Efficacy	0–29 years newly diagnosed AML	Induction 7+3 +/– GO and post-consolidation +/–GO	Completed	1070	III	GO improved EFS (3 years: 53.1% vs. 46.9%; *P* = 0.04) but not OS (3 years: 69.4% vs. 65.4%; *P* = 0.39)
		NCT00551460	Efficacy	Adult, older previously untreated HR APL	ATRA + GO + Arsenic	Completed	78	II	3 Years CR 74% (95% CI: 62 to 84%).
		NCT00895934	Efficacy and safety	50 years and older R/R AML	Vorinostat+Azacitidine+GO	Completed	52	I/II	ORR 41.9% (95% CI: 27.0–57.9%)
		NCT01409161	Efficacy and safety	10 years and older APL	Tretinoin and arsenic +/– GO	Recruiting	150	II	No results
		NCT03287128	Efficacy and safety	18 years and older R/R AML	GO 3 or 6 mg/m^2^	Recruiting	300	None	No results
		NCT03737955	Efficacy and safety	MRD in AML	Fractionated GO	Recruiting	36	II	No results
	SGN-CD33A	NCT01902329	Safety	AML	SGN-CD33A + Azacytidine/Decitabine	Completed	195	I	Recommended dose of SGN-CD33A is 40 μg/kg
	SGN-CD33A	NCT02785900	Efficacy	Older Newly Diagnosed AML	SGN-CD34A + Azacytidine/Decitabine	terminated	240	III	Due to safety; A higher deaths rate.
CD123	CSL360	NCT00401739	Safety and tolerability	R/R or HR AML	CSL360	Completed	40	I	No results
	CSL362	NCT01632852	Safety	CD123+ AML in remission	CSL362	Completed	30	I	No results
	CSL362 (JNJ-56022473)	NCT02472145	Efficacy and safety	AML ineligible for intensive chemotherapy	CSL362+ Decitabine	Completed	326	II/III	CR rate of experimental and control group was 16.6 and 11.9%, and OS was 5 and 7 months
PR1/HLA-A2	Hu8F4	NCT02530034	Safety	Advanced HM	Anti-PR1/HLA-A2 (Hu8F4)	Recruiting	60	I	No results
VEGF-C	Anti-VEGF-C	NCT01195506	Functions, mechanisms	AML	Anti-VEGF-C	Unknown	40	None	No results
FLT3	FLYSYN	NCT02789254	Safety, efficacy,	AML with MRD	Fc-optimized FLT3 Antibody	Recruiting	28	I/II	No results
KIR	IPH2101	NCT01256073	Safety and tolerability	60–80 years AML	Fully human anti-KIR antibody	Completed	21	I	No results

*AML, Acute Myeloid Leukemia; APL, Acute Promyelocytic Leukemia; CI, Confidence Interval; CR, Complete Remission; GO, Gemtuzumab Ozogamicin; HM, Hematologic Malignancies; MRD, Minimal Residual Disease; ORR, Overall response rate; R/R, relapsed/refractory*.

### Anti-CD20 Antibody

CD20, a non-glycosylated transmembrane phosphoproteinis, is a B-lineage specific antigen expressed on both normal and malignant cells during nearly all stages of B-cell differentiation, but not expressed on hematopoietic stem cells, pro-B cells and plasma cells (54). It has an important role in cell cycle progression, differentiation, and modulation of apoptosis pathways (55).

#### Rituximab

A chimeric antibody targeting CD20 containing a murine variable region and a human Fc region. In the study of Thomas et al. (56) to investigate the impact of addition of rituximab to the modified hyper-CVAD regimen, Hyper-CVAD combined with Rituximab could improve the outcome of young CD20 positive Ph(–) B-ALL patients, with the complete remission duration 70% vs. 38% in 3 years, OS 75% vs. 47% in 3 years, but it did not improve the survival of elderly patients (56). In a randomized Graall-R 2005 Study to evaluate the addition of rituximab to a conventional chemotherapy backbone in 209 patients ages 18 to 59 years with newly diagnosed CD20-positive Ph-negative B-cell ALL, results showed that the cumulative incidence of relapse (CIR) was decreased by Rituximab (2 years CIR 18% vs. 30.5%, *p* = 0.02), but OS was not longer (2 year OS, 71% vs. 64%; *p* = 0.095). Rituximab enhances the efficacy of chemotherapy without additive toxicity, but the improvement in the treatment of ALL is modest (57–59).

#### Ofatumumab

A second generation anti CD20 monoclonal antibody that binds to a site different than Rituximab. Ofatumumab is more effective than Rituximab in inducing cytotoxic mediated by ADCC and CDC. In 55 patients with newly diagnosed ALL and 4 patients with CR who underwent a median of 8 cycles of Ofatumumab, the results showed that with the exception of 1 patient, other patients (98%) achieved CR after the first cycle, and 53 patients (93%) obtained MRD negative, and 3 year CRD and OS were 78 and 68%, respectively. The OS of patients with CD20 < 20% and ≥20% in 3 years were 82 and 64%, respectively (*p* = 0.96) (60). It was confirmed to be safe and highly effective in patients with CD 20 positive ALL.

### Anti-CD22 Antibody

CD22 is expressed in early stages of B-cell development until terminal differentiation except for plasma cells and expressed in most (>90%) patients with B-cell ALL (61). When CD22 binds to the monoclonal antibody, antibody/antigen complex will be rapid internalization leading to antitumor effect (62).

#### Epratuzumab

An unconjugated human monoclonal antibody that binds to the third extracellular domain of CD22. The combination of CD 22 with Epratuzumab results in the direct phosphorylation of upstream inhibition receptors of BCR signaling (63). The tolerance of Epratuzumab combined with reinduction chemotherapy is good, but compared with chemotherapy alone, the rate of CR2 is not significantly improved. Although increasing the times of Epratuzumab, MRD still has no significant improvement (64). In the SWOG S0910 study, for adult r/r pre-B ALL, the total effective rate (CR + PR) of the adult r/ r pre-B ALL was 52% when Epratuzumab combined with clofarabine and cytarabine, significantly higher than the previous efficacy of clofarabine/cytarabine alone (17%) (65). In the multicenter prospective phase II study of Hyper-CVAD combined with Epratuzumab for young r/r CD22 pre-B ALL, almost half of the patients were MRD negative. It is expected to improve the survival of patients if it is used in first-line therapy especially before allogeneic hematopoietic stem cell transplantation because of Epratuzumab's ability to reduce MRD levels (66).

#### Inotuzumab Ozogamicin (InO)

A humanized anti-CD 22 ADC combined with calicheamicin, a cytotoxic antibiotic agent (67). After InO binds CD22 then internalizes itself rapidly, the conjugated calicheamicin was delivered intracellularly causing double-strand DNA cleavage and cell apoptosis. In a phase III clinical trial involving 326 r/r ALL patients to compare with InO and standard care, patients were randomly divided into InO group and standard intensive chemotherapy group (67). The complete remission rate in InO group was significantly higher than that in standard treatment group (80.7% [95% CI, 72.1–87.7] vs. 29.4% [95% CI, 21.0–38.8], *p* < 0.001). The MRD negative rate in InO group was significantly increased (78.4% vs. 28.1% *P* < 0.001). PFS in InO group was prolonged (median PFS: 5.0 months [95% CI, 3.7~5.6], 1.8 months [95% CI, 1.5~2.2]), and median OS was 7.7 months (95% CI, 6.0–9.2) vs. 6.7 months (95% CI, 4.9~8.3). InO significantly improved the remission rate in patients with higher CD22 levels (≥90%) and lower CD22 levels (<90%). It is worth noting that InO has a good response rate to both high and low tumor loads, while Blinatumomab has a relatively good response rate to patients with low tumor load.

### Anti-CD52 Antibody

CD52 is a glycoprotein linked to the cell membrane by a phosphatidylinositol glycan linkage, and is expressed on the surface of nearly all normal and malignant B-lymphocytes and T-lymphocytes, monocytes, and macrophages, but not on plasma cells and HSCs (68). It was reported CD52 played a role in cell lysis via ADCC, complement-mediated cell lysis, and possibly apoptosis (69).

#### Alemtuzumab

Alemtuzumab is a recombinant DNA-derived humanized monoclonal antibody that targets CD52. Binding to CD52 induces cell lysis through complement activation and ADCC (70). Single drug Alemtuzumab has limited effect on relapsed or refractory acute leukemia. In phase I study of CALGB, the use of Alemtuzumab after remission can eradicate MRD, but it is also associated with viral infection (71). Studies have shown that low-dose Alemtuzumab before transplant may efficiently prevent severe acute and chronic GVHD by its T-cell depleting effect (72, 73).

### Anti-CD19 Antibody

CD19 appears at early stages and persists through all stages of B-cell maturation and is homogeneously expressed on malignant cells. It is one of the important membrane antigens involved in the activation and proliferation of B cells.

#### SAR 3419

A humanized anti-CD 19 ADC that binds to a derivative of the potent microtubule-acting cytotoxic agent, Maytansin, which induces cell cycle arrest and apoptosis (74). In the study of xenotransplantation of CD19 preB ALL and mixed lineage leukemia (MLL) in mice, SAR3419 delayed the progression of the disease (75). However, in the multicenter, single-arm phase II clinical trial in patients with r/r ALL, only 4 of the 17 evaluable patients responded (ORR was 25.5%) and duration of response was only 1.9 months (76). Because of its unsatisfactory therapeutic response, the clinical trial was terminated earlier suggesting that SAR3419 monotherapy is not ideal for r/r ALL.

#### SGN-CD19A

A humanized ADC targeting CD 19 linked to microtubule interfering agent monomethyla-uristatin F, which inhibits microtubule assembly, triggers cell-cycle arrest of G2-M phase growth and induces cell apoptosis. In the study of exploring SGN-CD19A safety in the treatment of r/r B-ALL and Lymphoma, the phase I clinical trial study showed good tolerance to SGN-CD19A. The objective response rate was 30% in 33 patients who could be evaluated (NCT01786096).

#### SGN-CD19B

A highly active ADC proved by preclinical studies, targeting CD 19 based on Pyrrolidine diazepine, which releases DNA crosslinkers instead of microtubules inhibitors. *In vivo* experiments on monkeys, SGN-CD19B effectively cleaned CD20 B lymphocytes from peripheral blood and lymphoid tissues. Phase I clinical trials are under way to explore the therapeutic potential of SGN-CD19 in r/r B-NHL.

Selected trials of antibody directed therapy for ALL has been completed or is ongoing as shown in Table 2.

**Table 2:** Selected completed or ongoing trials of antibody directed therapy for ALL.

**Target**	**Biological**	**Clinical trials**	**Purpose**	**Patients**	**Intervention/ Treatment**	**Status**	***N***	**Phase**	**Results**
CD20	Rituximab (R)	NCT00427791	Efficacy	Up to 60 years ALL	Etoposide + TBI +/–R	Completed	23	II	Median PFS was 4.3 months for R group and 12.5 months for control group
		NCT00199004	Efficacy and safety	15–65 years standard risk ALL	Chemotherapy +R	Completed	60	IV	No results
		NCT01358253	Efficacy and safety	CD20+ adult ALL	Hyper CVAD+/–R	Completed	100	IV	No results
		NCT01085617	Efficacy	PreB-ALL	Chemotherapy +/– R	Recruiting	811	III	No results
	Ofatumumab	NCT02199184	Efficacy	Newly diagnosed or R/R Burkitt leukemia or ALL	DA-EPOCH + Ofatumumab	Recruiting	40	II	No results
CD22	Epratuzumab	NCT00098839	Efficacy	2–31 years relapsed CD22-positive ALL	Epratuzumab once weekly or twice weekly	Completed	134	II	For Epratuzumab once weekly or twice weekly, CR2 rate was 0.646 and 0.660, EFS rate at 4 months was 0.604 and 0.640. Rate of MRD <0.01% at the end of Block 1 was 0.195 and 0.295
	InO	NCT01564784	Efficacy	Adults R/R ALL	InO vs. standard chemotherapy	Completed	326	III	For InO group and standard-therapy group, CR rate was 80.7% vs. 29.4% (*P* < 0.001), median PFS was 5.0 months vs. 1.8 months (*P* < 0.001); median OS was 7.7 months vs. 6.7 months (*P* = 0.04)
		NCT03150693	Efficacy	18–39 years newly diagnosed preB- ALL	Frontline chemotherapy +/– InO	Recruiting	310	III	No results
		NCT03441061	Efficacy	18 years and older B-ALL with positive MRD	InO	Recruiting	40	II	No results
	Moxetumomab pasudotox	NCT00659425	Safety	6 months to 25 years R/R CD22+ ALL or NHL	Moxetumomab Pasudotox	Completed	57	I	Moxetumomab pasudotox was proved safety and activity in R/R ALL
CD52	Alemtuzumab	NCT00061048	Efficacy and safety	ATL	Alemtuzumab	Completed	29	II	Markedly additive antitumor activity
		NCT02689453	Efficacy and safety	18 years and older R/R chronic and acute ATL	IL-15 + Alemtuzumab	Recruiting	30	I	No results
CD19	SGN-CD19A	NCT01786096	Safety and tolerability	1 year and older B- ALL and highly aggressive lymphomas	SGN-CD19A once or twice every 21 days (0.3–6 mg/kg)	Completed	92	I	No results

*ALL, Acute lymphoblastic leukemia; ATL, Adult T-Cell Leukemia; EFS, Even;t-free Survival; MRD, Minimal Residual Disease; InO, inotuzumab ozogamicin; HR, hazard ratio; NHL, non-Hodgkin lymphoma; TBI, Total Body Irradiation; R/R, relapsed/refractory*.

## Bispecific Antibody/Trispecific Antibody

Bi- and trispecific antibodies, are single chain variable fragment (Scfv) consisting of at least two different specific antibodies one for tumor-associated surface antigens and the other for surface antigens on effector cells, such as CD3 ε on T cells or CD16 on NK cells. Through the double specificity of BiTE, the tumor cells were combined with T effector cells in HLA-independent manner.

### CD3/CD33 Bispecific T-Cell Engagers (BiTE)

#### AMG 330

CD3/CD33 BiTE antibody is dual specificity for CD3 and the sialic acid-binding lectin CD33. The cytotoxicity of AMG 330 to AML cells can be mediated not only by T cells, but also by killing CD33 MDSCs (77). In the presence of AML cells, AMG 330 specifically induced expression of CD69 and CD25 as well as release of IFN-γ, TNF, interleukin (IL)-2, IL-10, and IL-6 (78). AMG 330 can overcome some limitations of CD 33 targeted drugs. It neither regulates the expression of CD 33 nor is affected by the activity of ABC transporter. The density of target antigen, the dose of antibody and the ratio of E/T are the key factors to determine the effect of BiTE (79). The results showed that T cell ligands could regulate AMG 330, and inhibitory ligands PD-L1 and PD-L2 decreased the cytotoxicity of AMG 330, while the activated ligands CD 80 and CD 86 enhanced the cytotoxicity of AMG 330. The synergistic therapy with the regulation of T cell receptor signal can further enhance the effect of this targeted therapy (80, 81). The expression of CD 33 and splicing polymorphism are also associated with the cytotoxicity of AMG 330 *in vitro* (82). A phase I clinical study of AMG 330 for r/r AML is ongoing (NCT02520427).

#### AMG 673

An extended half-life BiTE. At present, there is no pre-clinical data report on AMG 673, but clinical research on r/r AML is being carried out (NCT03224819).

#### CD33/CD3 TandAbs

CD 33/CD3 directional tandem tetravalent bispecific antibody (TandAbs). These antibodies provide two binding sites for each antigen to maintain the affinity of a bivalent antibody. Its molecular weight exceeds the renal clearance threshold, so it has a longer half-life than the smaller antibody structure. This TandAbs can induce strong, dose-dependent cell lysis in CD 33 AML cells. This effect is regulated by the ratio of effector cells to target cells, and the existence of T cells is strictly required. The activation, proliferation and maximal AML cell lysis of T cells were related to the high affinity of CD 33 and CD3. High affinity TandAbs has a wide range of activity *in vitro* in primary or r/r AML patients. Delayed and inhibited tumor growth was observed in HL-60 xenotransplantation model of immunodeficient mice. CD 33/CD3 TandAbs is the potential of a new approach to the treatment of AML (83).

### CD16/CD33 Bispecific Killer Engagers (BiKE)/Bispecific Killer Engagers (TriKE)

#### CD16/CD33 BiKE

CD16/CD33 BiKE was also named “1633” which can induce the cytotoxicity of NK cells to human AML cells by eliminating MDSCs and reverse the MDSC mediated immunosuppression of NK cells (84, 85). When incubated with AML cells, CD16/CD33 BiKE is able to specifically trigger the cytotoxicity and cytokine release of NK cells. CD16/CD33 BiKE can overcome the autoinhibitory signal and effectively induce the inhibitory effect of NK cell effector on AML. *In vitro*, CD16/CD33 BiKE ± ADAM 17 inhibitor enhances the activation and specificity of NK cells to CD 33 (+) AML, and may be used for recurrent AML or post-transplant anti-leukemia therapy in the future (85).

#### 161533 TriKE

Although “1633” has anti-tumor effects, it lacks the ability to induce proliferation of NK cells. IL-15 plays an important role in the proliferation and activation of NK cells. Therefore, a Trike, including a modified IL-15 crosslinking agent between CD 16 and CD 33 single-chain Fv fragments (ScFvs), named “161533” was developed, this structure is capable of inducing NK cells proliferation and survival while allowing CD33-specific cytotoxic activity (86). Clinical trial of CD16/IL-15/CD33 Trikes in the treatment of high-risk heme malignancies is currently underway (NCT03214666).

### CD3/CD123 Dual-Affinity Re-Targeting (DART)

#### MGD006

##### CD3CD123-DART

DART is composed of two antigen-binding specific (A+B) heavy chains and light chain variable regions on two independent polypeptide chains (VLA-VHB-VLB-VHA), and is stabilized by another C-terminal bridge (87). MGD006 is a bispecific molecule that recognizes CD3 and CD123 membrane proteins, redirecting T cells to kill CD123-expressing cells, which can induce the activation and expansion of T cells and mediate the rapid clearance of AML (88). It kills AML cells and primary AML cells in a dose-dependent manner, and the safety and effectiveness are also verified in monkey (89, 90). Phase I clinical studies have been conducted on the safety of MGD006 in the treatment of r/r AML or intermediate-2/high-risk MDS (NCT02152956).

#### XmAb14045

A uniquely structured anti-CD123 T cell recruitment antibody developed by Xencor. XmAb technology ensures structural stability and prolongs serum half-life by retaining inactive FC fractions. The safety and toxicity of XmAb14045 were evaluated in patients with CD123+ hematologic malignancies in phase 1 I clinical study (NCT02730312).

### CD3/CLL1 BiTE

CLL-1 is highly expressed in AML blasts and LSCs. Unlike other targets such as CD33 and CD123, CLL-1 is not expressed on normal HSCs, which can ensure hematopoietic recovery (48). The double-specific CLL-1/CD3 antibody (MCLA-117), a full-length human IgG1 bispecific antibody, lacking Fc effector function and containing a common light chain, was developed. MCLA-117 is able to induce target antigen-specific cytotoxicity of allogeneic or autologous T cells to primary AML cells at low E:T ratio. Phase I clinical study incorporating all subtypes of adult r/r AML and newly diagnosed, high-risk cytogenetic elderly AML to verify the safety, toxicity and efficacy of MCLA-117(NCT03038230). Other studies on CLL1 BiTE, such as CD3/CLL1 TDB (T cell-dependent bispecific), a full length human IgG1 bispecific antibody engineered for improved pharmacokinetic and altered Fc-mediated functions could extended half-lives, have shown that it has good PK properties and antitumor activity, and has little effect on the non-target of HSC (48).

### CD3/CD19 BiTE

#### Blinatumomab (AMG103)

A BiTE immunotherapy with dual specificity for CD19 and CD3. Blinatumomab simultaneously binds CD3+ cytotoxic T cells and CD19+ B cells and redirects T cells to lyse malignant cells (91). First of all, the phase I clinical study of MRD positive B-ALL treated with Blinatumomab was carried out. The results showed that Blinatumomab had a good response to B-ALL regardless of MRD after hemotherapy (92). A subsequent phase II clinical study of r/r preB-ALL indicated that Blinatumomab significantly increased ORR and OS. After Blinatumomab 2 cycles, ORR was 69% and the median survival time was 9.8 months (93). In a multi-center, single-arm, phase II clinical study to observe the safety and efficacy of Blinatumomab in the treatment of adult r/r preB-ALL, the adverse reactions were mainly granulocytopenia fever and controlled neurotoxicity. The CR rate was 32% [95% CI, 26%~40%] and the median remission time was 6.7 months. About 31% (95% CI, 25%~39%) of the patients had MRD response (94). Based on the above research, FDA approved the application of Blinatumomab to Ph(–) r/r preB-ALL on December 3, 2014. There are many clinical studies on Blinatumomab, including the phase III clinical study of Blinatumomab for the first/second recurrence of ALL, the phase II clinical trial for recurrent Ph + ALL and the first-line treatment for MRD + ALL. In a study of 405 preB-ALL patients who received a large number of previous pretreatments, patients were randomly divided into single drug Blinatumomab group and standard chemotherapy group. The results showed that the median survival time of Blinatumomab group was significantly better than that of chemotherapy group (7.7 months vs. 4.0 months) (95). In the adult r/r preB ALL treated with Blinatumomab, the OS of the patients with MRD reaction was significantly longer than that of the patients without MRD reaction, and the T cell amplification was found to be larger in the long-term survivors. It suggests that the long-term survival of the patients may be related to the MRD reaction and the high expansion of T cells (96).

### CD3/CD20 BiTE

#### CD20-TDB

B cells targeting anti-CD20/CD3 T cell dependent bispecific antibody (CD20-TDB), is a full-length humanized immunoglobulin G1 molecule. In monkeys, CD20-TDB at a single dose of 1 mg/kg can effectively kill B cells in peripheral blood and lymphoid tissues and exhibit pharmacokinetic characteristics similar to those of conventional monoclonal antibodies (97).

### CiTE

One mechanism of limiting BiTE activity may be T cell incompetence and exhaustion driven by PD-1/PD-L1 and so on. Blocking the PD-1/PD-L1 pathway can increase the cytotoxicity of CD33/CD3 BiTE to leukemia cells (81). Herrmann et al. fused the PD-1 extracellular domain (PD-1EX) with CD3 × CD 33 BiTE to form CiTE. Studies have shown that CiTE with PD-1EX domain increases the specificity. Early clinical trials are under way. If preliminary trials prove to be free of severe immune-related adverse events, this could be an important step forward (98, 99).

Selected trials of bispecific or Tri-Specific T cell or killer Engagers antibody directed therapy for AML and ALL has been completed or is ongoing as shown in Table 3.

**Table 3:** Selected completed or ongoing trials of bispecific or Tri-Specific T cell or killer Engagers antibody directed therapy for AML and ALL.

**Target**	**Biological**	**Clinical trials**	**Purpose**	**Patients**	**Intervention/Treatment**	**Status**	***N***	**Phase**	**Results**
CD3/CD33	AMG 330	NCT02520427	Safety and tolerability	R/R AML	AMG330 0.5–960 μg/day infusion in cycles from 14 to 28 days	Recruiting	70	I	No results
	AMG 673	NCT03224819	Safety and tolerability	18 years and older R/R AML	AMG 673	Recruiting	50	I	No results
	JNJ-67371244	NCT03915379	Safety and tolerability	R/R AML or MDS	JNJ-67371244	Recruiting	90	I	No results
	CD33/CD3 TandAbs (AMV564)	NCT03144245	Safety and tolerability	18 years and older R/R AML	AMV564	Recruiting	148	I	No results
CD16/CD33	161533 TriKE	NCT03214666	Safety and tolerability	HR heme malignancies, R/R AML and advanced SMCD	161533 TriKE	Not yet recruiting	60	I/II	No results
CD3/CD123	MGD006	NCT02152956	Safety and tolerability	R/R AML or intermediate-2/high risk MDS	Flotetuzumab (MGD006)	Recruiting	179	I/II	No results
	XmAb14045	NCT02730312	Safety and tolerability	18 years and older CD123+ hematologic malignancies	XmAb14045	Recruiting	105	I	No results
	JNJ-63709178	NCT02715011	Safety and tolerability	18 years and older R/R AML	JNJ-63709178	Recruiting	60	I	No results
CD3/CLL1	MCLA-117	NCT03038230	Safety and tolerability	18 years and older AML	MCLA-117	Recruiting	50	I	No results
CD3/CD19	Blinatumomab	NCT02877303	Efficacy	Adults with B-ALL	Hyper-CVAD + Blinatcumomab as frontline therapy	Recruiting	60	II	No results
		NCT03982992	Efficacy, safety, and tolerability	18 years and older treatment-resistant mixed chimerism or MRD of preB-ALL after Allo-HSCT	Blinatumomab+ donor lymphocyte infusion	Recruiting	12	II	No results
		NCT01466179	Efficacy and safety	18 years and older Ph-, primary R/R leukemia	Blinatumomab	Completed	225	II	After two cycles Blinatumomab, CR+CRh rate was 43% (95% CI: 36–50)
		NCT01207388	Efficacy, safety, and tolerability	18 years and older ALL patients with MRD	Blinatumomab 15 μg/m^2^/day for 4 cycles	Completed	116	II	After 1 cycle of Blinatumomab, complete MRD response was 78%
		NCT02000427	Efficacy	18 years and older Ph+ ALL R/R to TKI	Blinatumomab	Completed	45	II	36% (95% CI, 22 to 51%) achieved CR/CRh after the first two cycles

*ALL, Acute lymphoblastic leukemia; Allo-HSCT, Allogeneic hemopoietic Stem Cell Transplantation; AML, Acute Myeloid Leukemia; HR, High Risk; MDS, Myelodysplastic Syndrome; MRD, minimal residual disease; Ph(–), Philadelphia-chromosome negative; R/R, relapsed/refractory; SMCD, Systemic mast cell disease (Systemic Mastocytosis)*.

## Chimeric Antigen Receptor (CARs)

CARs are genetically engineered cell membrane binding receptors which can activate T cells by linking from the extracellular antigen binding region to the intracellular signal domain via the spacer, whose effect on target antigen is independent of MHC (100). Genetic material encoding the chimeric antigen receptor is transferred into the patient's T cells using transfection, gamma retroviral or lentiviral recombinant vectors, or a transposon system (101–104). The advantages and limitations of each approach have not been fully elucidated recently. CARs contain an extracellular tumor antigen-recognizing scFv linked to an intracellular signaling component comprised of T-cell receptor (TCR) primary domain and often encompassing additional co-stimulatory endodomains (e.g., CD28, CD137 (4-1BB), CD134 (OX40), CD27, ICOS, etc.) (105). CAR transmits a signal by its ligand to the intracellular T cells through a signaling domain, typically the CD3-Zeta chain. The incorporation of co-stimulatory molecules can augment the effects of zeta chain signaling and hence enhance T cell proliferation and persistence (106). According to co-stimulators in signal transduction domain, CAR could be divided into four generations. The first-generation CAR T cells lacked co-stimulatory molecules and had little anti tumor activity (107). The second-generation CARs contain one co-stimulatory signaling domain CD28 or 4-1BB and the third-generation CARs contain two co-stimulatory signaling domains, with the first consisting of a CD28 or 4-1BB domain and the second provided by other molecules, such as OX40, CD28, or 4-1BB (105). Whether third generation CARs benefit more than second generation CARs remains unknown (108). Recent preclinical results indicate that CD137-based co-stimulatory domains are better than those based on CD28 at preventing T cell exhaustion (109). In recent years, the fourth-generation “armored CAR” T cells are engineered to additionally express cytokines or co-stimulatory ligands, aimed at enhancing expansion and longevity of the CAR T cells (110). Optimization of CARs design is an effective way to improve anti-tumor effect.

### CD33 CART

#### CD33 CART

Wang et al. demonstrated that CD33 CAR-T cells exhibited potent antileukemic efficacy, and CAR-T cells with 4-1BB co-stimulatory ligands performed better in antileukemic function than with CD28. Further analysis showed that CD33 4-1BBz.CAR-T cells had an increased central memory compartment and showed resistance to exhaustion (111). In NSG mice model of AML xenotransplantation, a novel second-generation anti-CD33 CAR that incorporates a 4-1BB-CD3ζ signaling tail, CD 33 CART can significantly reduce tumor load and prolong survival time (112). But it also causes toxic effects on normal hematopoietic cells. In order to reduce the toxicity, SS Kenderian et al. designed a transient expression of mRNA anti-CD33 CAR (113). Recently published in Cell, using genetic engineering to inactivate the CD33 gene of HSCs can avoid the toxic effect of CD33 CART on bone marrow, thus making it possible for CD33 CART to treat AML (114).

### CD 123 CART

CD 123 CART and CD 123-CAR cytokines induced killer cells (in which PBMC is activated by interferon γ and IL-2) have become a potential therapy for AML (115, 116). It is worth noting that CD 123-CART on normal hematopoietic function is smaller than that of CD33 CART. Modification of anti-CD123 scFv with VH and VL chains of different monoclonal antibodies can reduce the bone marrow toxicity of AML mice (117). The first clinical trial of CART-123 for r/r AML was conducted in our country in 2016 (NCT02937103), and there are other two trials ongoing (NCT03556982)/ (NCT03672851). Stage I clinical trials of CD123-CART for recurrent AML after Allo-HSCT are also underway (NCT03114670).

### CLL1 CART

#### CLL1 CART

CLL-1 CART cells generated by Zhou using the scFv region of the mAb against CLL-1 coupled to the co-stimulatory domains of CD28, 4-1BB, and the CD3-ζ chain could specifically dissolve CLL-1 cells and primary AML cells *in vitro* (118). Anti-tumor activity was also observed in AML mice and the survival time of mice was prolonged. Importantly, CLL-1 CART can specifically clear CLL-1 myeloid progenitor cells and mature myeloid cells, whereas normal HSCs are unaffected by their lack of CLL-1 expression (118, 119).

Other potential target antigens for AML include CD44v6 (120), FR β (121), NKG2D (122), and PR1/HLA-A2 (53), and so on.

### CD19 CART

#### CD19 CART

The CR rate of r/r ALL treated with CART19 was as high as 70–90%, and a sustained remission was observed without additional treatment (123–125). In order to improve the efficacy, the main focus is on the design of CAR. Phase I trials of 19–28z CART in 16 patients with r/r ALL showed a total CR rate of 88%, enabling most patients to transition to allo-HSCT (126). The safety and long-term results of 53 adult patients with relapsed ALL treated with 19–28z CART were evaluated. Fourteen cases developed severe cytokine release syndrome (26%) and 1 case died. Eighty three percentage of the patients achieved complete remission. Median follow-up was 29 months (range 1–65 months), median EFS was 6.1 months (95% CI, 5.0–11.5 months) and median OS was 12.9 months (95% CI, 8.7–23.4 months). In patients with low tumor load, the median OS was 20.1 months, and the incidence of cytokine release syndrome and neurotoxic events was significantly lower than that in patients with high tumor load (127). The third generation CART19 1928zT2 designed by Li Peng also achieved a good effect in the treatment of relapsed ALL (128). Besides, 4-1BB co-stimulatory signal is also engineered with the use of lentiviral-vector technology for gene transfer and permanent T-cell modification, named CTL019. After 30 patients with r/r ALL received CTL019 treatment, 27 cases (90%) received CR. The 6 months EFS was 67% (95% CI, 51~88) and OS was 78% (95% CI, 65~95) (61). Another study of 29 patients with r/r B-ALL treated with CD 19 4-1BB co-stimulated CART cells concluded the 86% MRD negative rate (129). FDA has granted CTL 019 breakthrough treatment status.

### Compound CART

The compound of CART: 123b-33bc CAR T, targeting CD 123 and CD 33 at the same time, not only eliminate the tumor load of AML, but also prevent the recurrence caused by the escape of antigen or the persistence of LSC. It has strong antitumor activity *in vivo* (130). With regard to CD123/CLL1 CART, phase II clinical trials plan to recruit 20 patients with r/r AML younger than 70 years old to verify their safety and toxicity.

According to research, it shows that PD-1 inhibitor can regulate the CART cell responses, suggesting that the PD-1 pathway may be critical for determining the T-cell immunotherapy response of CAR-modified cells. The combination of PD-1 inhibitor and CART is except to improve the treatment response and remission time.

Selected trials of CAR cell therapy for AML and ALL has been completed or is ongoing as shown in Table 4.

**Table 4:** Selected completed or ongoing trials of CAR cell therapy for AML and ALL.

**Target**	**Biological**	**Clinical trials**	**Purpose**	**Patients**	**Intervention/Treatment**	**Status**	***N***	**Phase**	**Results**
CD33	CD33-CAR-T	NCT03126864	Safety and tolerability	1–80 years CD33+ R/R AML	CD33-CAR-T cell infusion	Recruiting	39	I	No results
CD123	UCART123	NCT03190278	Safety and activity	R/R AML, and newly diagnosed HR AML	UCART123	Recruiting	162	I	No results
	CD123 CAR/28,EGFRt+ T cells	NCT02159495	Safety and tolerability	R/R AML and persistent/recurrent BPDCN	Autologous or allogeneic CD123+ CAR T cells	Recruiting	42	I	No results
Compound CAR	CLL1-CD33 CART	NCT03795779	Safety and tolerability	R/R HR hematologic malignancies.	CLL1-CD33 compound CAR T cells	Recruiting	20	I	No results
	CD123/CLL1 CART	NCT03631576	Safety and tolerability	R/R AML.	CD123/CLL1 compound CAR-T Cells	Recruiting	20	II/III	No results
CD19	CD19 CAR/137 T cells	NCT02030847	Efficacy and safety	R/R B-ALL	Single infusion of autologous CD19 CAR T cells	Completed	42	II	CR+CRi was 60% at Day 28 after infusion
	huCART19	NCT03792633	Efficacy	1–29 years VHR B-ALL	huCART19 infusion	Recruiting	85	II	No results
	CARCIK-CD19	NCT03389035	Safety and tolerability	1–75 years R/R B-ALL After HSCT	CARCIK-CD19 (Allogeneic CIK cells transduced with a transposon CD19 CAR gene)	Recruiting	18	I/II	No results
	CD19 CAR/137 T cells	NCT02965092	Efficacy and safety	R/R B-cell Malignancies	CAR-T cells	Recruiting	80	I/II	No results
	KTE-C19	NCT02625480	Safety and efficacy	2–21 years R/R preB-ALL	Fludarabine and Cyclophosphamide followed infusion of KTE-C19	Recruiting	100	I/II	No results
	CD19.CAR/28 T cells, CD19.CAR/28.137 T cells	NCT01853631	Safety and efficacy	Advanced B-NHL, ALL and CLL	CD19.CAR/28 T cells and CD19.CAR/28.137 T cells +/– Cyclophosphamide and Fludarabine	Recruiting	64	I	No results
	CD19 CART	NCT02146924	Safety and efficacy	18 years and older high-risk CD19+ ALL	1.CD19CAR-CD28-CD3zeta-EGFRt-expressing Tcm-enriched T-lymphocytes; 2.CD19CAR-CD28-CD3zeta-EGFRt-expressing Tn/mem-enriched T-lymphocytes	Recruiting	88	I	No results
CD20	CD20 CART	NCT02710149	Safety and efficacy	14–75 years B cell malignancies	CD20-targeted CAR-T cells	Recruiting	45	I/II	No results
	CD20/CD22/CD10-CART	NCT03407859	Safety and efficacy	18–60 years R/R B-ALL	Sequential treatment with CD20/CD22/CD10-CART after CD19-CART treatment	Recruiting	30	I	No results
CD22	CD22 CART	NCT03262298	Safety and efficacy	18–65 years B cell malignancies	Anti-CD22-CAR-transduced T cells	Recruiting	20	I/II	No results
Compound CAR	CD19 and CD20 or CD22 CART	NCT03398967	Safety and efficacy	R/R leukemia and lymphoma	Universal dual specificity CD19 and CD20 or CD22 CAR-T cells	Recruiting	80	I/II	No results
	CD19/20-CART	NCT03097770	Safety and efficacy	R/R B-cell leukemias and lymphomas	Anti-CD19/20-CAR vector-transduced T cells	Recruiting	20	None	No results
	AUTO3	NCT03289455	Safety and activity	1–24 years R/R B-ALL	AUTO3 (CD19/22 CAR T cells)	Recruiting	50	I/II	No results

*AML, Acute Myeloid Leukemia; ALL, Acute lymphoblastic leukemia; BPDCN, Blastic Plasmacytoid Dendritic Cell Neoplasm; CAR, Chimeric Antigen Receptor; CIK, Cytokine Induced Killer; CLL, Chronic Lymphocytic Leukemia; HR, High-Risk; R/R, relapsed/refractory; HSCT, Hematopoietic Stem Cell Transplantation; NHL, Non-Hodgkin's Lymphoma; R/R, relapsed/refractory; Tcm, central memory T cells; Tn/mem, naive and memory T cells; VHR, Very High-Risk*.

## Immune Checkpoint Inhibitor and Immune Agonist

The two main immune checkpoint blocking pathways in clinical studies are cytotoxic T lymphocyte antigen (CTLA-4) and programmed cell death pathway. Programmed cell death pathways include programmed cell death protein 1 (PD-1) and its ligands, programmed death ligands 1 (PD-L1) and 2 (PD-L2). Although most clinical studies of blocking PD-1 and CTLA-4 with humanized monoclonal antibodies are directed at solid tumors and lymphoma, PD-1 and CTLA-4 also play a role in leukemia, GVL and GVHD (131). Blocking CTLA-4 and blocking PD-1/PD-L1 pathway with anti PD-L1 antibody enhanced the anti-leukemia immune response in mice (131, 132). Combined blocking of PD1/PD-L1 and Tim-3/galectin-9 may help to prevent CD8 (+) T cell failure in patients with advanced AML and other malignant hematological diseases (133). Studies have shown that epigenetic drugs can regulate the expression of immunological checkpoint molecules on TIL and tumor cells (134), such as Azacytidine upregulating the expression of PD-1 and PD-L1 in MDS/AML. The up-regulation of these genes may be related to the emergence of Azacytidine resistance and the decrease of survival time (135). To improve response rates, epigenetic drugs combined with PD-1/PDL-1 inhibitors are also being tested in clinical trials (NCT02397720, NCT02530463). The timing of immune checkpoint blocking may be more important for the occurrence of hematological malignancies, especially leukemia, because the tumor itself often destroys the host's immune function. Due to high tumor burden and proliferation rate in acute leukemia, the disease may develop before checkpoint antibodies (especially when used alone) have sufficient time to activate the immune response. Therefore, when there is minimal residual disease and a complete immune system, the application of immune checkpoint inhibitors may achieve the best results. A number of clinical trials are currently underway to use checkpoint antibodies as a single drug or in combination with standard chemotherapy regimens to treat newly diagnosed and relapsed leukemia patients.

OX40, a member of the tumor necrosis factor receptor family, is an activating receptor expressed on the surface of activated CD4+ T cells and CD8+ T cells. OX40 signaling activates downstream NF-κB and PI3K pathways, whose sustained activation prolongs T cell survival and promotes the activation and proliferation of T cells. OX40 also inhibits the differentiation and activity of T-reg cell and improves immunosuppressive effects thereby to reinforce antitumor immunity. Besides, OX40L signal transduction promotes NK cells activation, cytokines production, cytotoxicity and the like (136–138). OX40/OX40L has received widespread attention as a potential target for immune activation. Clinical trial of OX40 antibody to treat leukemia is ongoing (NCT03390296, NCT03410901). However, OX40 is expressed on leukemic blasts in a substantial percentage of patients with AML, and so after stimulation with OX40 agonist, OX40 can mediate proliferation and release of cytokines that act as growth and survival factors for the leukemic cells (136). Therefore, the therapeutic effect of OX40 antibody on leukemia needs further verification.

Selected trials of Immune Checkpoint Inhibitor therapy for AML and ALL has been completed or is ongoing as shown in Table 5.

**Table 5:** Selected completed or ongoing trials of Immune Checkpoint Inhibitor therapy for AML and ALL.

**Target**	**Biological**	**Clinical trials**	**Purpose**	**Patients**	**Intervention/Treatment**	**Status**	***N***	**Phase**	**Results**
PD-1	Nivolumab	NCT02532231	Efficacy	AML in remission at high risk for relapse	Nivolumab	Recruiting	30	II	No results
		NCT02275533	Efficacy	AML patients after chemotherapy	Nivolumab once every 2 weeks repeatedly every 2 weeks for 46 courses	Recruiting	80	II	No results
	Pembrolizumab	NCT03969446	Safety and efficacy	Newly-diagnosed OR R/R AML or MDS	Pembrolizumab and Decitabine	Not yet recruiting	54	I	No results
		NCT02845297	Safety and efficacy	R/R AML and in newly diagnosed older AML	Azacitidine and Pembrolizumab	Recruiting	40	II	No results
		NCT03286114	Efficacy	Relapse of primary malignancy after Allo-HSCT	Pembrolizumab	Recruiting	20	I	No results
		NCT02708641	Efficacy	Patients ≥60 AML not transplantation candidates	Post-remission treatment with Pembrolizumab 200 mg once every 3 weeks	Recruiting	40	II	No results
		NCT02767934	Safety and efficacy	ALL With MRD	Pembrolizumab	Recruiting	21	II	No results
PD-L1	Durvalumab	NCT02775903	Efficacy and safety	Previously untreated HR MDS or in elderly (> = 65 years) AML not eligible for HSCT	Azacitidine+/– Durvalumab	Active, not recruiting	213	II	No results
CTLA4	Lpilimumab	NCT01757639	Safety and tolerability	AML With MRD	lpilimumab	Completed	42	I	No results
		NCT02890329	Safety and tolerability	R/R MDS/AML	lpilimumab and Decitabine	Recruiting	48	I	No results
CD47 PD-L1	Hu5F9-G4 Atezolizumab	NCT03922477	Safety and pharmacokinetics	R/R AML	Hu5F9-G4 and Atezolizumab	Not yet recruiting	21	I	No results
CTLA4 PD-1	Ipilimumab Nivolumab	NCT03600155	Safety and tolerability	HR or R/R AML	lpilimumab and Nivolumab	Recruiting	55	I	No results
		NCT02397720	Safety and efficacy	R/R or newly diagnosed AML	Azacitidine, Ipilimumab, Nivolumab	Recruiting	182	II	No results
PD-1 TIM-3	PDR001 MBG453	NCT03066648	Safety and tolerability	AML or HR MDS	Decitabine, PDR001, MBG453	Recruiting	175	I	No results
PD-1 CD3/CD19	Pembrolizumab and Blinatumomab	NCT03512405	Safety and Efficacy	R/R ALL	Pembrolizumab and Blinatumomab	Recruiting	36	I/II	No results
OX40	PF-04518600	NCT03390296	Safety and tolerability	R/R AML	PF-04518600, Avelumab Azacitidine, Glasdegib, Venetoclax, GO	Recruiting	18	I	No results

*ALL, Acute lymphoblastic leukemia; AML, Acute Myeloid Leukemia; GO, Gemtuzumab Ozogamicin; HR, High-Risk; MDS, Myelodysplastic Syndrome; PDR001, a high-affinity, ligand-blocking, humanized IgG4 monoclonal antibody directed against PD-1; MBG453, a high-affinity, humanized anti-TIM-3 IgG4 monoclonal antibody which blocks the binding of TIM-3 to phosphatidylserine; HSCT, Hematopoietic Stem Cell Transplantation. R/R, relapsed/refractory*.

## NK Cells

NK cells can exert direct cytotoxic activity without antigen-specific initiation and activate other immune cells to promote anti-leukemia immune response (139). The activity of NK cells in patients with acute leukemia is affected by a number of mechanisms, including reduced expression of activated receptors and decreased cytokine secretion (140, 141). In addition, the low expression of NK ligands in leukemia cells and the production of soluble immunosuppressive factor are also the reasons of impaired NK cells activity (142, 143). NK cell-based tumor immunotherapy strategies include anti KIR antibodies, cytokines (including IL-15 and IL-2), adoptive HLA- haploidentical NK cells and use of NK cell lines (139, 144–146). Other strategies include reducing Treg cells and using bispecific antibodies, such as CD16 on NK cells and leukemia cell-specific antigens, which significantly enhance the cytotoxicity mediated by antibody dependent cells, one of the main mechanisms for eliminating leukemic cells.

IL-2 diphtheria toxin fusion protein (IL2DT) can enhance the clearance of AML by haplotype NK cells. After 7 days of NK cells adoptive infusion, the depletion of Treg and the persistence of NK cells predicted a good clinical response. IL2DT could reduce the host Treg cells and then improve the efficacy of haploid NK cells in the treatment of AML (147). Cytokine induced memory like NK cells enhance its cytotoxicity to myeloid leukemia, and this effect is independent of KIR, which has been confirmed in NSG transplanted tumor model mice (148). Recent studies have shown that ATO enhances the cytotoxicity of NK cells, and the mechanism may be that ATO can change the receptor and ligand structure of NK cells (149). Samudio et al. used oncolytic virus HSV to activate peripheral blood mononuclear cells to lyse leukemia cells. This activation depends, at least in part, on TLR-2, and acts primarily by activating NK cells (150). Fc-optimized CD133 antibody (293 C3-SDIE) significantly enhanced the activation, degranulation and lysis of primary CD133-positive AML cells by allogeneic and autologous NK cells, thereby promoting the clearance of AML cells by NK cells (151). Williams et al. found that CD16 NK-92 cells combined with an antibody against leukemia stem cell antigen could improve the survival of AML model mice (44).

Selected trials of NK cells therapy and other immunotherapies for AML and ALL has been completed or is ongoing as shown in Table 6.

**Table 6:** Selected completed or ongoing trials of NK cells therapy and other immunotherapies for AML and ALL.

**Target**	**Biological**	**Clinical trials**	**Purpose**	**Patients**	**Intervention/Treatment**	**Status**	***N***	**Phase**	**Results**
NK	NK Cells	NCT01904136	Safety, tolerability	High-risk myeloid malignancies undergoing HSCT	Allo-HSCT /BMT/chemotherapy/NK cell therapy/TBI	Recruiting	90	I/II	No results
	CIML NK cell	NCT02782546	Safety and efficacy	AML	Cytokine induced memory-like NK cell adoptive therapy after haploidentical donor hematopoietic cell transplantation	Recruiting	60	II	No results
	NK cells	NCT02185781	Safety, tolerability	Ph+ ALL in CHR but with P/R MRD ≥60 years or not eligible for other post-CHR treatment modalities	Enriched and expanded autologous NK cells infusions	Recruiting	6	I	No results
	NK cells	NCT00995137	Safety, tolerability	Up to 18 years B-ALL	Genetically modified haploidentical natural killer cell infusions	Completed	14	I	No results
	NK cells	NCT01787474	Safety and efficacy	18 years and older R/R AML	IL-21-expanded natural killer cells	Recruiting	44	I/II	No results
Vaccine	DEC-205/NY-ESO-1 fusion protein CDX-1401	NCT03358719	Safety and efficacy	MDS or Low Blast Count AML	DEC-205/NY-ESO-1 fusion protein CDX-1401/Decitabine/Nivolumab/Poly ICLC	Recruiting	18	I	No results

*ALL, Acute lymphoblastic leukemia; Allo-HSCT, Allogeneic Hematopoietic Stem Cell Transplantation; AML, Acute Myeloid Leukemia; BMT, Bone Marrow Transplantation; CHR, Complete Hematologic Remission; HSCT, Hematopoietic Stem Cell Transplantation; NK cells, Natural Killer Cells; P/R, Persistent/Recurrent; R/R, relapsed/refractory; TBI, Total-B*.

## Conclusions

The use of immunotherapy in the treatment of acute leukemia has greatly improved the choice of treatment. It not only provides an opportunity for hematopoietic stem cell transplantation, but also increases the MRD response rate, reduces the MRD level, and further improves the remission time and survival time. Combined use of multiple immunotherapies, such as checkpoint inhibitors combined with other immunotherapy, can improve efficacy and duration of response. Although immunotherapy exhibits a good prospect, its toxicity deserves attention. The side effects of immunotherapy on normal HSCs are urgent problems to be solved, and more precise targets need to be studied to reduce the damage to normal cells.

## Author Contributions

FZ and YS designed the study. YS collected data and wrote the manuscript. All authors read and approved the final manuscript.

### Conflict of Interest Statement

The authors declare that the research was conducted in the absence of any commercial or financial relationships that could be construed as a potential conflict of interest.
